# ERK Signaling Regulates Light-Induced Gene Expression via D-Box Enhancers in a Differential, Wavelength-Dependent Manner

**DOI:** 10.1371/journal.pone.0067858

**Published:** 2013-06-26

**Authors:** Philipp Mracek, Cristina Pagano, Nadine Fröhlich, M. Laura Idda, Ines H. Cuesta, Jose Fernando Lopez-Olmeda, F. Javier Sánchez-Vázquez, Daniela Vallone, Nicholas S. Foulkes

**Affiliations:** 1 Institute of Toxicology and Genetics, Karlsruhe Institute of Technology, Eggenstein, Germany; 2 Department of Physiology, Faculty of Biology, Regional Campus of International Excellence “Campus Mare Nostrum”, University of Murcia, Murcia, Spain; Kent State University, United States of America

## Abstract

The day-night and seasonal cycles are dominated by regular changes in the intensity as well as spectral composition of sunlight. In aquatic environments the spectrum of sunlight is also strongly affected by the depth and quality of water. During evolution, organisms have adopted various key strategies in order to adapt to these changes, including the development of clocks and photoreceptor mechanisms. These mechanisms enable the detection and anticipation of regular changes in lighting conditions and thereby direct an appropriate physiological response. In teleosts, a growing body of evidence points to most cell types possessing complex photoreceptive systems. However, our understanding of precisely how these systems are regulated and in turn dictate changes in gene expression remains incomplete. In this manuscript we attempt to unravel this complexity by comparing the effects of two specific wavelengths of light upon signal transduction and gene expression regulatory mechanisms in zebrafish cells. We reveal a significant difference in the kinetics of light-induced gene expression upon blue and red light exposure. Importantly, both red and blue light-induced gene expression relies upon D-box enhancer promoter elements. Using pharmacological and genetic approaches we demonstrate that the ERK/MAPK pathway acts as a negative regulator of blue but not red light activated transcription. Thus, we reveal that D-box-driven gene expression is regulated via ERK/MAPK signaling in a strongly wavelength-dependent manner.

## Introduction

The day-night cycle dominates the environment and poses many challenges for the survival of plants and animals. As well as regular cycles of light intensity, the spectrum of sunlight also exhibits daily changes as a result of the relative position of the sun in the sky [1], [2]. For example at twilight, as well as a reduced overall light intensity the sun's rays pass through a thicker layer of the atmosphere. As a result there is a relative enrichment of blue-green wavelengths, the so-called Chappuis effect [2], [3]. Changes in the spectrum of sunlight are accentuated in aquatic environments because water acts as a filter for specific wavelengths as a function of its depth and quality [4]. Generally, shorter visible wavelengths of light (blue/violet) penetrate deeper through water than longer wavelengths (red/orange).

Organisms have adopted various evolutionary strategies to adapt to regular changes in sunlight. One key strategy has been to develop timing mechanisms, notably the circadian clock that enables the anticipation of the day-night cycle. At the core of this highly conserved mechanism is a regulatory network composed of interlocking transcription translation feedback loops. In the vertebrate clock, positive elements (CLOCK and BMAL) activate the transcription of negative elements *period* (*per*) and *cryptochrome* (*cry*) [5] which in turn inhibit the action of the CLOCK:BMAL complex, thus closing the feedback loop [6]. Characteristically this mechanism does not complete one cycle in precisely 24 hours. Therefore it needs to be reset on a daily basis by environmental signals that are indicative of the time of day, so-called zeitgebers [7], [1]. The most powerful zeitgeber is light and so most organisms have evolved specialized light detection mechanisms that regulate elements of the core clock machinery.

A second key strategy to adapt to the day night cycle has been to develop mechanisms driven by direct exposure to sunlight. This includes for example the repair of UV-induced DNA damage where the activity of many elements is directly activated upon exposure to visible light. Thus, DNA photolyase enzymatic activity is directly light dependent [8] and in many organisms, the transcription of DNA photolyase genes is upregulated by visible wavelengths of light [9], [10], [11].

Both strategies rely upon light-dependent signal transduction systems. However to date, our understanding of the precise nature of these photoresponsive mechanisms remains very much incomplete. Teleosts and notably zebrafish have been established as attractive vertebrate models for studying how light regulates gene expression [12], [13]. As in other vertebrates, most zebrafish tissues contain independent “peripheral” circadian clocks [14], [15]. However, while in mammals light entrainment of peripheral clocks occurs indirectly via the retina and the “central” clock of the suprachiasmatic nucleus (SCN) [16], in zebrafish the peripheral clocks are entrained by direct exposure to light [17], [18]. Direct illumination of zebrafish cells activates the expression of a subset of clock genes. This in turn leads to circadian clock entrainment [19], [20], [21]. Furthermore, the expression of many other non-clock related genes such as those involved in DNA damage repair are known to be light inducible [22], [23]. We have demonstrated that the D-box enhancer element in the promoters of light inducible genes serves as the primary promoter element directing light-driven transcription [24], [25].

To date, the precise identity of the peripheral photoreceptors remains unclear. One report studying light-regulated mRNA expression of the clock gene *per2*, concluded that blue light sensing cryptochromes were the principal photoreceptors [26]. However, flavin-containing oxidases have also been implicated as peripheral blue light photoreceptors in zebrafish [21]. Recent studies have revealed an impressive diversity of opsin genes in fish species, many of which are expressed in peripheral tissues [2], [27], [28], [29]. In a recent comparative study involving zebrafish and the cavefish *Phreatichthys andruzzii* [30], we demonstrated that TMT-opsin and Opn4m2 (Melanopsin, Opn4.1 according to ZFIN nomenclature) serve as peripheral tissue photoreceptors and that both respond preferentially to blue/green (468 nm/530 nm) but not to red light (657 nm). However, we also revealed that red light is able to induce clock gene expression in zebrafish cells predicting the existence of additional red light sensing photoreceptive mechanisms [30]. The precise nature of the signal transduction mechanisms linking these various photoreceptors with changes in gene expression remains very much uncertain although light induced changes in ERK signaling have been reported in zebrafish cells [26], [31]. Together, these findings predict that co-expression of multiple photoreceptors in zebrafish peripheral tissues may enable a complex gene expression response to light.

In this manuscript we attempt to unravel this complexity by comparing the effects of two specific wavelengths of light upon signal transduction and gene expression regulatory mechanisms. By using pharmacological and genetic approaches we show that D-box driven gene expression is regulated via ERK/MAPK signaling in a strongly wavelength-dependent manner.

## Results

### Light-induced genes are differentially activated by blue and red light

**Figure 1:**
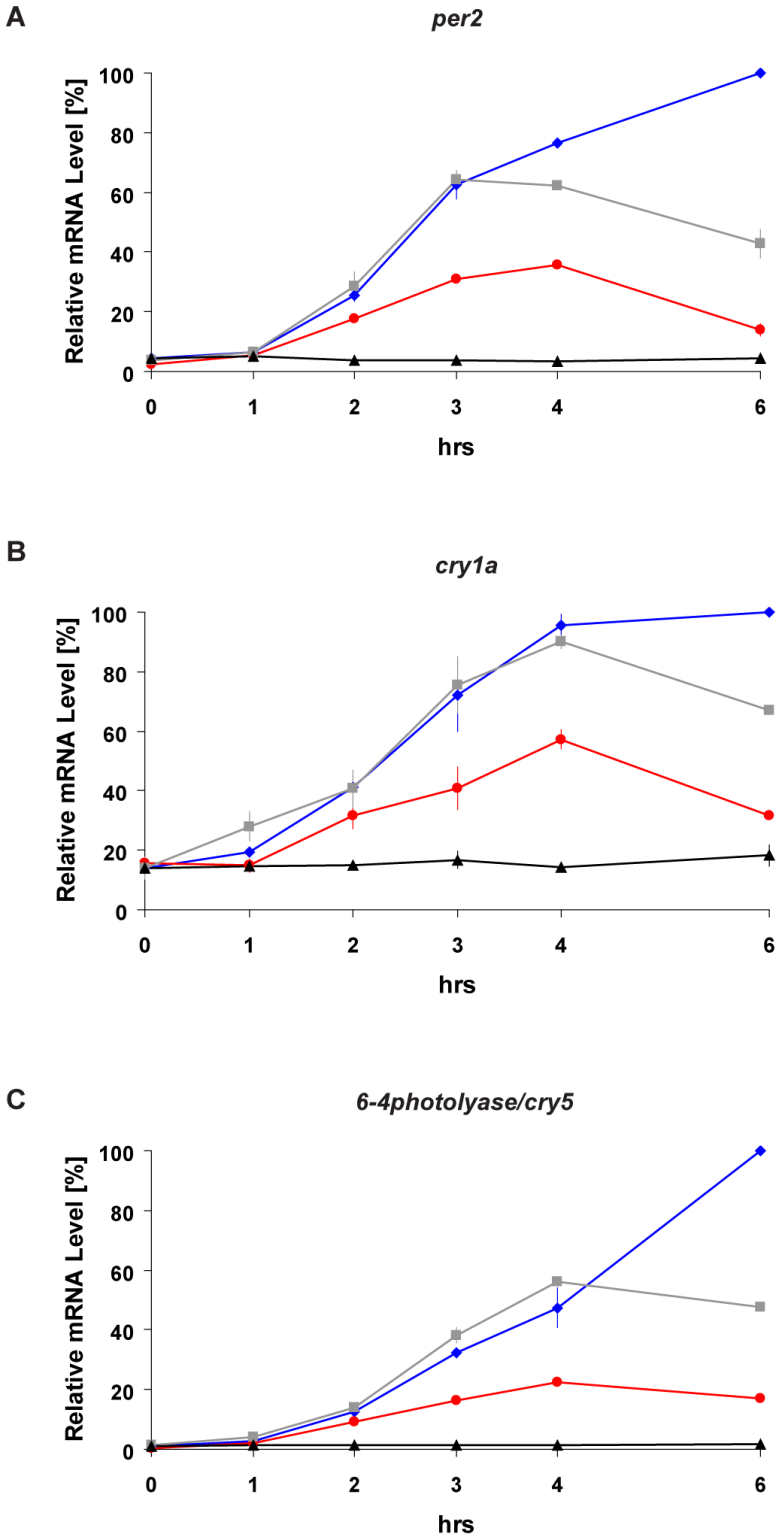
Activation of light-inducible genes by blue, red and white light. (A–C) Quantitative RT-PCR analysis (qRT-PCR) of light inducible genes in PAC-2 cells during constant darkness conditions (DD controls, black traces), white (grey traces), blue (blue traces) or red (red traces) light exposure. Total RNA was extracted from cells that were maintained in DD (control) or illuminated with the various light sources for different time periods (0, 1, 2, 3, 4, 6 hrs). Each gene is indicated above its respective panel. Relative mRNA levels (%) are plotted on the y-axis where the highest value measured during each experiment (Blue light exposure for 6 hours) is set as 100%. Time (hrs) is plotted on the x-axes. In each panel, points are plotted as the means of three independent experiments +/−SD. The results of statistical analysis are presented in Figure S1A–B.

We have previously shown that both blue and red light exposure can induce reporter gene expression in zebrafish peripheral tissues [30]. In addition it was reported that blue light was more effective in inducing expression of the clock gene *per2* in the Z3 cell line [26]. However, other light-induced genes were not examined. Thus, we first aimed to characterize the gene expression response to blue and red light in more detail. We exposed zebrafish PAC-2 cells to either white, blue (468 nm) or red (657 nm) light sources adjusted to deliver the same photon flux (1.42×10^18^±0.04×10^18^ photons/s/m^2^). We then measured the mRNA expression of three well studied light inducible genes, the clock genes *per2*, *cry1a* and the DNA repair enzyme gene *6-4 photolyase/cry5*, during 6 hours of light exposure (Figure 1). For all three genes, exposure to each of the light sources resulted in a significant up-regulation of expression compared with constant dark controls (black traces). Red (red traces) and white (grey traces) light lead to a peak of expression around 3–4 hours and a subsequent decline. Differently, blue light induces gene expression (blue traces) for the entire period of light exposure leading to significantly higher levels of transcripts than with red or white light at 6 hrs of light exposure (Figure 1 and Figure S1A–B for statistical analysis). Thus the kinetics of light induced gene expression appears to be wavelength dependent.

### Differential regulation by blue and red light is mediated through D-box promoter elements

**Figure 2:**
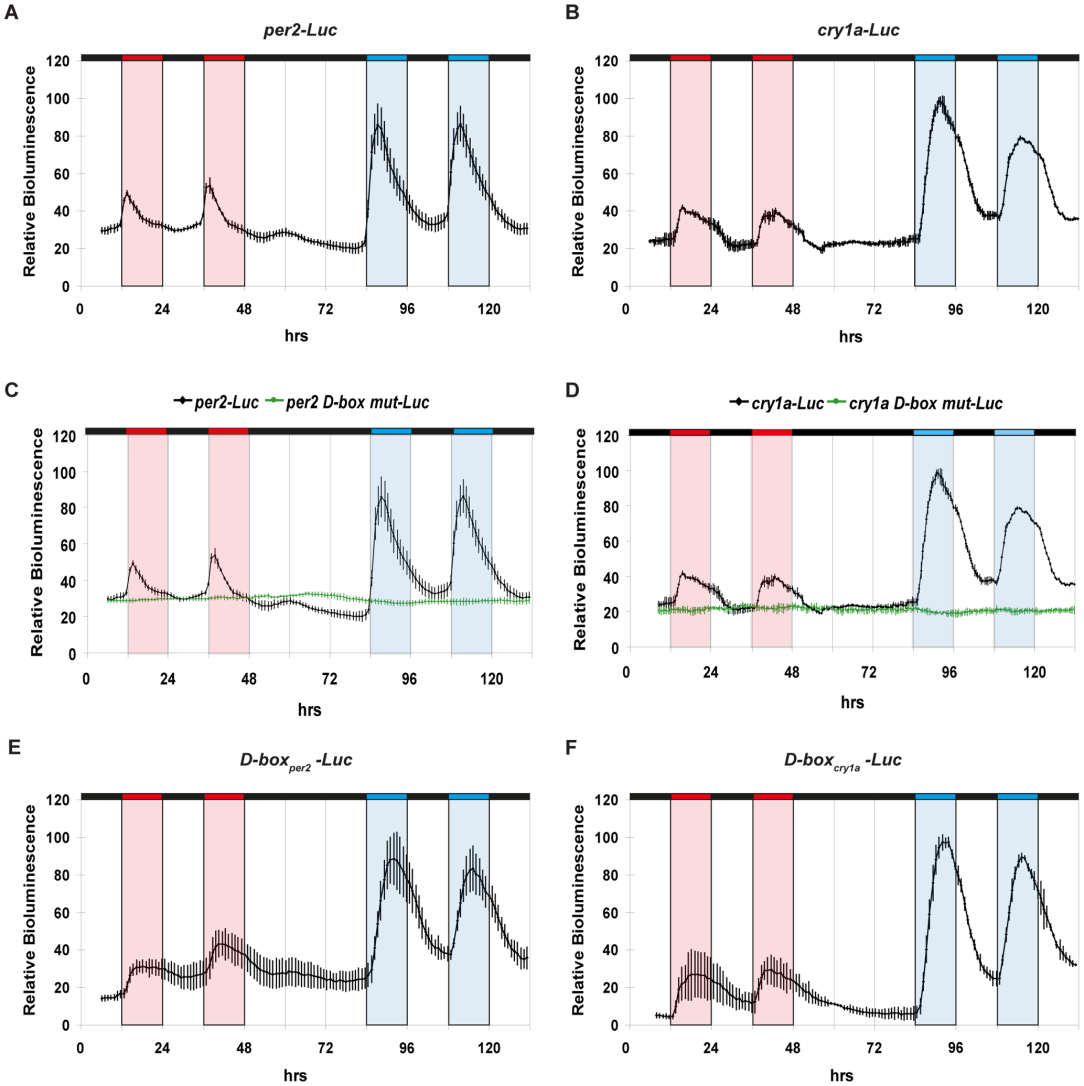
Differential regulation by blue and red light is mediated through D-box enhancer elements. (A–F) Real time bioluminescence assays of transfected PAC-2 cells. Each construct is indicated above its respective panel. In each panel relative bioluminescence is plotted on the y-axis and time (hrs) on the x-axis. Each time-point represents the mean of three independent experiments +/− SD. Red, blue and black bars above each panel represent the red light, blue light and dark periods, respectively. For clarity, blue, red and white background shading also indicates the blue, red and dark periods respectively. The results of statistical analysis are presented in Figure S1C–E.

Is this differential response of endogenous gene expression to different wavelengths of light based on a transcriptional mechanism? To directly address this question, we examined the effect of exposure to blue and red light upon luciferase reporter expression driven by two light inducible gene promoters (*per2-Luc and cry1a-Luc*) in zebrafish cells (Figure 2 A–B). Specifically we tested luciferase reporter expression during exposure to red and then blue light dark (LD) cycles (12 hours light∶12 hours dark), separated by a period of constant darkness. The intermediate period of constant darkness in the lighting regime was chosen in order to visualize any possible circadian clock regulation of these promoters following entrainment by red or blue light cycles. Specifically, clock-regulated rhythmic expression should persist in constant darkness. Consistent with endogenous gene expression, both *per2-Luc* and *cry1a-Luc* were more strongly activated by blue than by red light (Figure 2A–B and Figure S1C). Furthermore, a significantly earlier peak of expression was observed under red light compared with blue light exposure (Figure S1D). Finally, the absence of cycling gene expression following transfer from red or blue LD cycles to constant darkness confirms that the changes in reporter expression are purely light-driven (Figure 2A–B). We have previously identified D-box enhancers as the key promoter elements mediating white light-induced gene expression [24], [25]. Do these enhancers contribute to the observed differential gene expression response to blue and red light? We tested expression of the *per2-Luc* and *cry1a-Luc* constructs where the functional D-box elements had been disrupted by mutagenesis. Cells transfected with both *per2 D-box mut-Luc* and *cry1a D-box mut-Luc* were not induced either by blue or red light (green traces, Figures 2C–D) compared with wild type reporter construct controls (black traces) (Figure S1E for statistical analysis). These results show that activation of gene expression by both red and blue light is mediated exclusively by D-box enhancer elements. Are the D-box enhancer elements sufficient to mediate the differential red/blue light response? To address this question we examined the expression of heterologous reporter constructs based on multimerized D-box elements isolated from the *per2* or *cry1a* promoters (Figure 2 E–F). Similar to *per2-Luc* and *cry1a-Luc*, both *D-box_per2_-Luc* and *D-box_cry1a_-Luc* reporters were induced more strongly under blue light than red light (Figure S1C for statistical analysis). Furthermore, both heterologous reporters showed an earlier peak of expression in red than in blue light (Figure S1D for statistical analysis) and no cycling upon transfer to constant darkness conditions. Together, our data show that the D-box enhancer element is necessary and sufficient to mediate the differential transcriptional induction by blue and red light.

### ERK/MAPK signaling pathway differentially regulates light–induced gene expression

**Figure 3:**
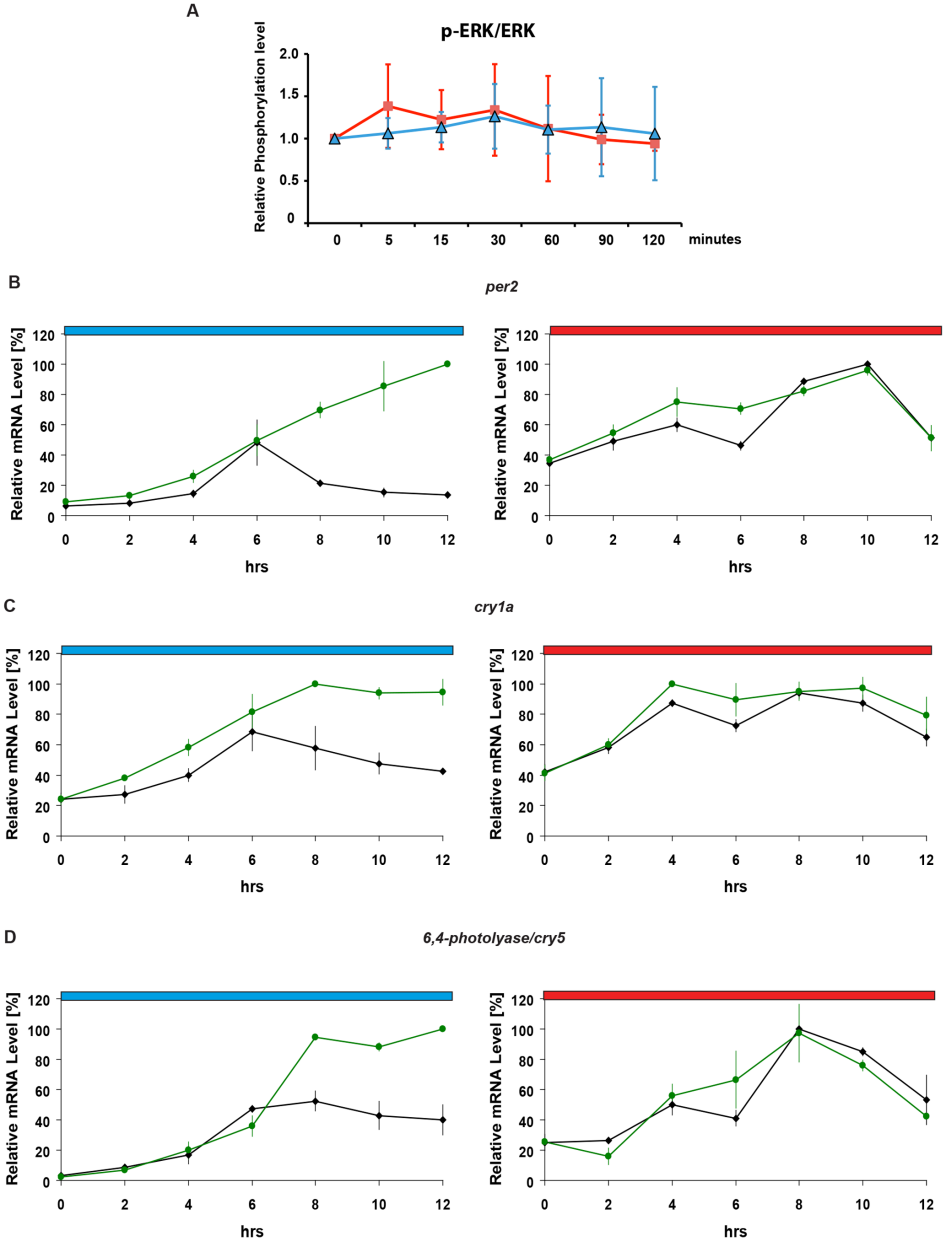
Tonic ERK/MAPK signaling down-regulates blue light-induced gene expression. (A) Graphical representation of relative phospho-ERK (p-ERK/ERK) levels under blue- (blue trace) and red-light (red trace) normalized using total ERK expression. On the y-axis relative phosphorylation levels are plotted with time point 0 min being set arbitrarily as 1. Time (min) is plotted on the x-axis. Each point represents the mean of six independent experiments +/−SD. No significant phospho-ERK induction was observed under either red (t-test p>0.05) or blue light (t-test p>0.05) at any time point analysed. Representative autoradiograph images are shown in Figure S2A. (B–D) qRT-PCR analysis of light inducible gene expression in the presence (green traces) or absence (black traces) of U0126 during 12 hours of blue or red light exposure. Cells were treated with either U0126 (1 µM) or DMSO as a control, 1 h before light exposure. Each gene is indicated above its respective panels. Blue and red bars above each panel indicate the wavelengths of light used. Relative mRNA levels (%) are plotted on the y-axes where the highest value measured during each experiment (each panel) is set as 100%. Time (hrs) is plotted on the x-axes. Each point represents the mean of three independent experiments +/−SD. The results of statistical analysis are presented in Figure S1F.

We next explored which signal transduction mechanisms may contribute to this differential transcriptional response. The ERK/MAPK signaling pathway has been previously implicated as a regulator of white light-induced gene expression [32], [26], [31]. Previous reports have documented increases in phospho-ERK levels upon exposure of zebrafish cells to white light (31). Thus, we tested whether dynamic changes of phospho-ERK levels might underlie the differential regulation by blue and red light. We exposed cells for 2 hours to either blue or red light and then ERK and phospho-ERK levels were analyzed by western blotting. No significant changes in phospho-ERK levels were detected either upon blue (p>0.05, t-test) or red light exposure (p>0.05, t-test) at any time point analysed (Figure 3A and Figure S2A) despite the induction of *per2* and *cry1a* expression observed under the same lighting conditions (Figure 1). Likewise, phospho-ERK levels also remained relatively constant during the exposure of cells to LD cycles (Figure S2B). These results indicate that either the ERK/MAPK signaling pathway is not involved in mediating red and blue light-induced gene expression or alternatively, there exists a differential ERK/MAPK regulation that does not entail significant changes in phospho-ERK levels.

To distinguish between these two possibilities, we used a pharmacological approach to suppress ERK/MAPK signaling. The MEK-specific inhibitor U0126 was previously reported to block white light-induced gene expression [26], [31]. Cells were treated with 1 µM U0126 during exposure to blue or red light and then mRNA expression of light inducible genes was analyzed. This dose was selected as the minimal concentration required for the repression of basal phospho-ERK levels by 80–90% (Figure S3). Upon U0126 treatment, no significant change in red light induced gene expression was observed (Figure 3B–D, right hand panels) (Figure S1F for statistical analysis). In contrast, following 6 hours of blue light exposure, a stronger and more sustained activation of light induced gene expression was observed compared with DMSO treated controls (Figure 3B–D, left hand panels) (Figure S1F for statistical analysis). Thus, these results point to a specific negative regulation by ERK/MAPK signaling in blue but not red light-induced gene expression.

**Figure 4:**
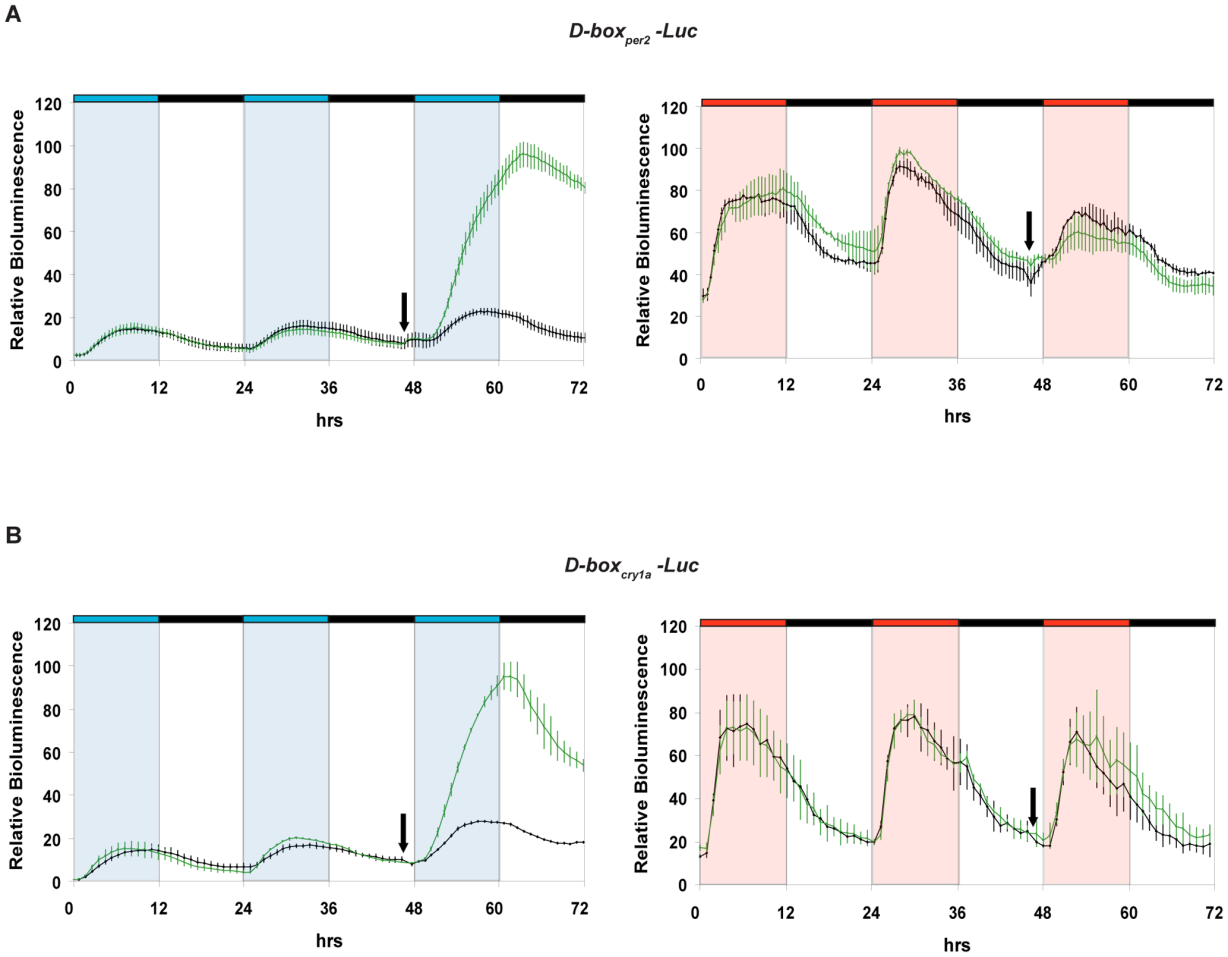
Blocking of the ERK/MAPK signaling pathway enhances blue light-induced reporter gene expression via D-box elements. Real time bioluminescence assays of PAC-2 cells transfected with the following constructs: (A) *D-boxper2-Luc*. (B) *D-boxcry1a-Luc*. All transfected constructs are in the presence (green traces) or absence (black traces) of 1 µM U0126. The black arrows indicate the start of treatments. In each panel relative bioluminescence is plotted on the y-axis and time (hrs) on the x-axis. Each time-point represents the mean of three independent experiments +/−SD. The results of statistical analysis are presented in Figure S1G. Blue, red and black bars above each panel represent the different lighting conditions. For clarity, blue, red and white background also indicates the blue, red and dark periods, respectively. See also Figure S3 for further experimental details.

Is the D-box enhancer element directly targeted by the blue light-dependent ERK/MAPK signal? We examined the effect of U0126 treatment upon the two heterologous luciferase D-box constructs (*D-box_per2_-Luc* and *D-box_cry1a_-Luc*) under blue light exposure (Figure 4 and Figure S3). For both constructs, following treatment with U0126, a stronger activation was observed under blue light compared with vehicle-treated controls. Confirming the wavelength-dependence of the ERK/MAPK signal, no effect of U0126 treatment was observed on red light induced reporter gene activation (Figure S1G for statistical analysis). Thus the D-box appears to mediate the inducing effects of U0126 treatment upon blue light-induced gene expression.

### ERK/MAPK signaling pathway negatively regulates blue light–induced gene expression

**Figure 5:**
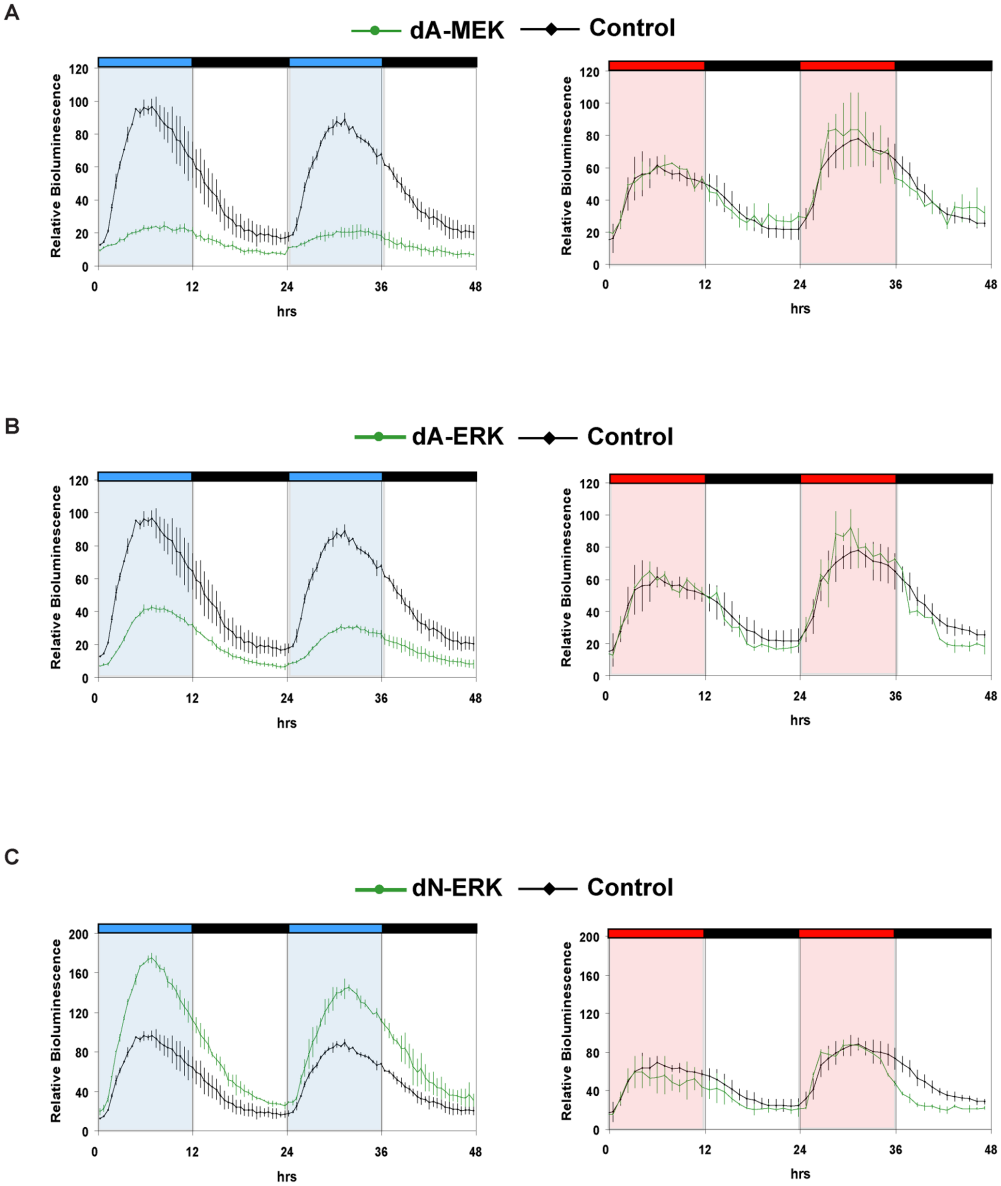
Impact of dominant active and dominant negative forms of MEK and ERK on D-box driven expression. Real time bioluminescence assays of PAC-2 cells co-transfected with *D-boxcry1a-Luc* and either *dA-MEK* (A), *dA-ERK* (B), *dN-ERK* (C) (green traces) or an empty expression vector control (A–C, black traces). In each panel, relative bioluminescence (%) is plotted on the y-axis where the highest value measured in the control (reporter construct alone) cells during each experiment is set as 100% and time (hrs) is plotted on the x-axis. Each time-point represents the mean of three independent experiments +/−SD. The results of statistical analysis are presented in Figure S1H. Blue, red and black bars above each panel represent the lighting conditions. For clarity, blue, red and white background also indicates the blue, red and dark periods, respectively. See Figure S4 for characterization of the various recombinant MAPK constructs.

To more stringently test the involvement of the ERK/MAPK pathway in blue light-induced, D-box mediated gene expression, we used a genetic approach based on either a dominant negative form of ERK or dominant active forms of ERK and MEK kinases (see Figure S4 for the controls of their expression and functionality). We transfected these expression constructs together with the *D-box_cry1a_-Luc* reporter into PAC-2 cells. Under blue light illumination, consistent with our previous results using U0126, expression of the dominant negative form of ERK (dN-ERK) resulted in a stronger upregulation of the D-box reporter compared with controls (Figure 5 and Figure S1H for statistical analysis). Furthermore, expression of the constitutively active MEK (dA-MEK) or ERK (dA-ERK) significantly repressed reporter gene induction (Figure 5 and Figure S1H for statistical analysis). Thus, we confirm that upon blue light exposure, the ERK/MAPK pathway serves to negatively regulate D-box driven transcription. In contrast, red light induced reporter gene expression was unaffected by co-expression of either the constitutively active or dominant negative kinases pointing to an ERK/MAPK independent mechanism mediating red light-induced D-box regulated transcription.

## Discussion

By comparing the effects of illuminating zebrafish cells with red and blue light we have revealed key differential responses at the levels of signal transduction and gene expression. We demonstrate that blue light exposure strongly induces gene expression via a mechanism that involves negative regulation by the ERK/MAPK pathway. In contrast, red light induced gene expression seems to be independent of ERK-MAPK signaling. Thus, our results predict the existence of additional light-regulated elements that positively regulate blue light induced gene expression as well as other signals that direct red light activation. We conclude that exposure to light can trigger multiple signaling pathways in a wavelength dependent manner that ultimately converge at the level of regulating gene expression.

Our results using the zebrafish PAC-2 cell line demonstrate that Illumination using either red or blue light is not associated with significant changes in overall phospho-ERK levels. What is the functional significance of a tonic negative regulatory signal in blue light driven transcription? Given the kinetics of blue light-induced gene expression upon pharmacological and genetic manipulation of the MAPK pathway, it is tempting to speculate that this might represent a mechanism whereby the amplitude or duration of induced gene expression is controlled by ERK/MAPK signaling.

Previous reports in zebrafish cells have portrayed ERK serving as a positive regulator of light-induced gene expression [26], [31]. We speculate that the different results we have observed relate in part to the fact that previous studies have employed different zebrafish cell lines, brighter “white light” sources with non-defined spectral compositions as well as higher doses of the MEK inhibitor U0126. We used a lower dose of this inhibitor that was selected experimentally as the minimal concentration required for the repression of basal phospho-ERK levels by 80–90% (see Figure S3 and [35]). Furthermore, importantly our results are also supported by the genetic approach of using dominant active and negative forms of the ERK and MEK kinases. Interestingly, in the mammalian SCN it has been well documented that light-induced phospho-ERK serves as a positive regulator of clock gene expression [36]. However, how the SCN clock and zebrafish peripheral clocks are regulated by light differs fundamentally. In the case of the SCN, light signals are conveyed indirectly from the retina via neural pathways and the activation of NMDA receptors [36]. In contrast, zebrafish cells perceive light directly via opsin photoreceptors that signal to regulate clock gene expression [30].

Here we show that in zebrafish cells the D-box promoter element is a regulatory target for both red and blue light signaling. In addition, D-boxes drive light induced expression of a wide range of genes in zebrafish cells [22], [25]. Thus, our results reinforce a growing body of evidence that the D-box represents a fundamental convergence point for light-driven signaling in teleosts. This is fundamentally different from the mammalian SCN where the transcription factor CREB via CRE enhancer elements, serves as the principal nuclear target for photic regulation of clock gene expression [36]. We have previously demonstrated the existence of a family of 12 D-box binding transcription factors of the PAR/E4BP4 family in zebrafish which exhibit diverse expression patterns in developing embryonic tissues [33]. This complex array of transcriptional regulators may contribute to integrating input from multiple light-activated signal transduction pathways. An important future challenge will be to systematically map protein modifications induced by specific wavelengths of light and assess their consequence for D-box enhancer function.

We observed that blue light exposure results in a stronger activation of gene expression than red light. An enhanced sensitivity to blue light could provide an adaptive advantage for fish generally, since it is these wavelengths that penetrate the deepest through water. This may account for the involvement of multiple blue light sensing mechanisms in fish cells [12]. However, the natural habitats of zebrafish are relatively shallow bodies of water [34] and so this species would also be naturally exposed to longer wavelengths of light [4]. From a more general perspective, teleosts inhabit a great diversity of environments where lighting conditions may vary considerably. The precise composition of the sunlight spectrum will vary as a function of depth and quality of water as well as being affected by the time of day and the seasons. Given our results, we predict that a key role of multiple photoreceptive mechanisms in fish may well be to integrate this complex and dynamic light input and thereby direct an optimal and appropriate cellular response. Thus, a diverse “toolbox” of photoreceptive mechanisms in fish that are able to respond to the full visible light spectrum may have contributed to the special ability of fish to colonize diverse aquatic habitats during evolution.

## Materials and Methods

### Cell Culture

The PAC-2 cell line [37] was cultured as previously described [18], [38]. For incubation under different lighting regimes, cells were maintained in thermostatically controlled darkrooms or light-sealed incubators. Illumination by monochromatic light sources was achieved using light-emitting diodes (LED, Kopa) as described elsewhere [30]. U0126 treatment was performed as recommended by the manufacturer (Sigma Aldrich). The dose of U0126 used (1 µM) was experimentally determined as recommended elsewhere (see Figure S3 and [35]). Transfections were performed using the FuGene HD reagent according to the manufacturer's protocol (Roche Diagnostics).

### Quantitative RT-PCR (qRT-PCR)

Total RNA was extracted using Trizol reagent (GIBCO-BRL) according to the manufacturer's instructions. Total RNA was reverse-transcribed into cDNA using Superscript III Reverse Transcriptase (Invitrogen) with a mixture of oligo dT and random primers. qRT-PCR analysis was performed using a StepOnePlus Real-Time RT-PCR System (Applied Biosystems) and SYBR Green I fluorescent dye (Qiagen). Relative expression levels were normalized using zebrafish β*-actin* mRNA. The relative levels of mRNA were calculated using the 2- ΔΔCT method. For each gene the primer sequences are listed in Table S1.

### Clock Gene Promoter Luciferase Reporter Constructs

All promoter constructs were based on the promoterless luciferase expression vector pGL3Basic (Promega). *cry1a-Luc* contains the 186 bp light responsive region identified in the *cry1a* promoter [25]. *cry1a D-box mut-Luc* contains the same *cry1a* light responsive region where the functional D-box is disrupted by site directed mutagenesis [25]. *per2-Luc* contains the 430 bp *per2* minimal promoter region [24] while in *per2 D-box mut-Luc* the functional D-box is disrupted [24] within the same minimal promoter region. All heterologous promoter reporter constructs were based on the minimal promoter luciferase reporter vector pLucMCS (Stratagene). *D-box_cry1a_-Luc* and *D-box_per2_-Luc* contain multimerized copies of the *cry1a* D-box 5′–AAGTTATACAAC–3′ [25] and *per2* D-box 5′–CTTATGTAAA–3′ [24] respectively. Finally, the ***AP1-Luc*** reporter construct contains four multimerized copies of the sequence 5′-TGACTCA-3′ [25].

### Expression Constructs

All dominant active and dominant negative ERK/MEK expression constructs were based on the CMV promoter driven expression vector pcDNA3.1 (Invitrogen) and have been described elsewhere [39], [40]. *dA-MEK* contains the dominant active form of hMEK1 [39]. *dA-ERK* consists of a dominant active form of the rERK2-hMEK1 fusion protein [40]. *dN-ERK* consists of a dominant negative form of the rERK2-hMEK1 fusion protein [40]. Functionality or the expression of these constructs in zebrafish cells was confirmed (Figure S4).

### Real-Time Bioluminescence Assay and Data Analysis

All real-time bioluminescence assays were performed and analyzed as described previously [18], [24] using an EnVision multilabel counter (Perkin Elmer) under various lighting conditions.

### Western blotting

Protein extracts were prepared by homogenizing samples in NP40 buffer including a cocktail of phosphatase inhibitors (Sigma Aldrich). The samples were electrophoresed on a SDS polyacrylamide gel and transferred to an Immobilon-P membrane (Millipore). Binding of the antibodies was visualized using the Pierce-ECL detection system (Thermo Scientific). Phospho-ERK, ERK and MEK antibodies were purchased from Cell Signaling. Phospho-ERK levels were all normalized using total ERK expression. In addition, to normalize for sample loading we used Vinculin (Sigma Aldrich) or α-Tubulin (Cell signaling) as a loading control. Autoradiographic images were quantified with the aid of ImageJ software.

### Statistical Analysis

Data were analyzed by unpaired t-test and two-way ANOVA using GraphPad Prism 4.0 for Windows (Graph Pad Software, http://www.graphpad.com). All the results were expressed as means +/−SD. p<0.05 was considered statistically significant. Peak time values were calculated using Ritme software (Antoni Diez-Noguera, University of Barcelona). The results of statistical analysis are presented either in Figure S1, in the results section or in the corresponding Figure legends.

## Supporting Information

Figure S1 **(A–H) Summary of statistical analysis.** The panels represent the results of statistical analysis for all experiments. The relevant figure and the type of statistical test performed are indicated above each panel. A colour code illustrates the type of illumination used and the names of genes, constructs or treatments are indicated in each panel. Statistically significant was considered as p<0.05. No Significant difference is indicated by N. S. In panel D, peak time values were calculated using Ritme software (Antoni Diez-Noguera, University of Barcelona) and n.d. indicates “not-determined” peak values.
(TIF)

Figure S2 **Western blot analysis of phospho-ERK under blue and red light.** Representative western blots of endogenous ERK (ERK), phospho-ERK (p-ERK) and Vinculin or α-Tubulin in PAC-2 cells during (A) 2 hours of either blue or red light exposure and (B) exposure for 36 hours to a 12 hours blue; 12 hours dark LD cycle. In panel A, the duration of light exposure of each sample is indicated in minutes (mins). These blots are representative of six independent experiments and the final quantification is presented graphically in Figure 3A. In panel B, the time points are indicated as zeitgeber times (ZT, where ZT0 represents lights on and ZT12 represents lights off). Note that the p-ERK and ERK antibodies both recognize the two forms of ERK (p42 and p44). Thus, with these antibodies two bands are more or less well resolved depending on the duration of electrophoresis. Levels of Vinculin or α-Tubulin were used as loading controls for each western blot. Blue and red bars above or below each blot represent the wavelengths and duration of light exposure while black bars indicate darkness. (C) Quantification of relative phospho-ERK levels from three independent experiments performed as in panel B. No significant circadian oscillation was observed as tested by Cosinor analysis (COSINOR v3.0.2 software, Antoni Diez-Noguera, University of Barcelona).
(TIF)

Figure S3 **Inhibition of ERK phosphorylation by U0126 treatment.** (A–B) Real time bioluminescence assays of PAC-2 cells transfected with *D-boxcry1a-Luc* and treated with a range of different U0126 concentrations or a DMSO control. The color code for each treatment is depicted in the key for the panels. The black arrows indicate the start of treatments. Relative bioluminescence is plotted on the y-axis and time (hrs) on the x-axis. Each time-point represents the mean of three independent experiments. Blue and black bars above the panels represent the different lighting conditions. For clarity, blue and white background also indicates the blue light and dark periods, respectively. (C) Representative western blots of endogenous phospho-ERK (p-ERK) and ERK levels in PAC-2 cells following 1 hr, 3 hrs, 12 hrs or 24 hrs of incubation with 1 µM U0126 or DMSO (control) and under DD conditions. (D) Real time bioluminescence assays of PAC-2 cells transfected with *D-boxcry1a-Luc*, in the presence (green trace) or absence (black trace) of the selected dose of U0126 (1 µM). The black arrow indicates the start of 48 hours of U0126 treatment. Relative bioluminescence is plotted on the y-axis and time from the first exposure to blue light (hrs) on the x-axis. Each time-point represents the mean of three independent experiments +/−SD. Periods of exposure to darkness or blue light are indicated as described for panels A and B.
(TIF)

Figure S4 **Characterization of dominant active and dominant negative ERK and MEK.** (A) Representative western blot of endogenous ERK phosphorylation (p-ERK) levels in PAC-2 cells sampled at the same time point each day during the third and fourth day after transfection with *dA-MEK* (+) or with an empty expression vector (−) (control). Western blot analysis of MEK, ERK and α-tubulin in the same extracts are also shown as controls. Note that in the presence of *dA-MEK*, the level of immunoreactive MEK protein is increased since the electrophoretic mobility of the recombinant and endogenous MEK proteins are identical (see materials and methods for details of the recombinant MEK protein). (B) Quantification of the western analysis in panel A, performed in triplicate with phospho-ERK values normalized using endogenous ERK levels. The dark and light grey bars represent phospho-ERK levels in the presence and absence (control) of *dA-MEK* respectively. Relative ERK phosphorylation levels are plotted on the y-axis where levels in the control samples were set arbitrarily as 1. Values are plotted as the means of three independent experiments +/−SD. The levels of phospho-ERK are significantly increased in the presence of the dA-MEK form for both days (p<0.001, t-test). (C) The expression of the ERK-MEK fusion-proteins produced by *dA-ERK* and *dN-ERK* in cell extracts prepared at the same time each day, from the second to the sixth day following transfection was detected by western blot analysis using phospho-ERK (p-ERK) antibodies. (Note the high molecular weight of these fusion proteins with respect to the endogenous phospho-ERK protein visible in the same membrane, see materials and methods for precise details). As a loading control, the membrane was also incubated with an anti α-tubulin antibody. Note that by the sixth day, less concentrated protein extracts were recovered due to decreasing viability of the transfected cells. (D) Real time bioluminescence assays of PAC-2 cells transfected with *AP1-Luc* in the presence of dA-MEK-1 (blue trace), dA-ERK-2 (green trace) and dN-ERK-2 (red trace). Control cells were transfected with the *AP1-luc* reporter and empty expression vector (black trace). The AP1 enhancer represents a well-documented nuclear target of MAPK signaling [41]. Bioluminescence (cps) was measured during five days after transfection and is plotted on the y-axis while time after transfection (hrs) is plotted on the x-axis. Each time-point represents the mean of three independent experiments. This data demonstrates the presence of functionally active dA-MEK and dA-ERK proteins during the entire time course of our experiments.
(TIF)

Table S1 **qRT-PCR primer sequences.**
(DOC)
