# Supplementary figures and images for: ERK Signaling Regulates Light-Induced Gene Expression via D-Box Enhancers in a Differential, Wavelength-Dependent Manner
Source: PLoS One. 2013 Jun 26;8(6):e67858. doi: 10.1371/journal.pone.0067858 (PMC3694018; doi:10.1371/journal.pone.0067858)

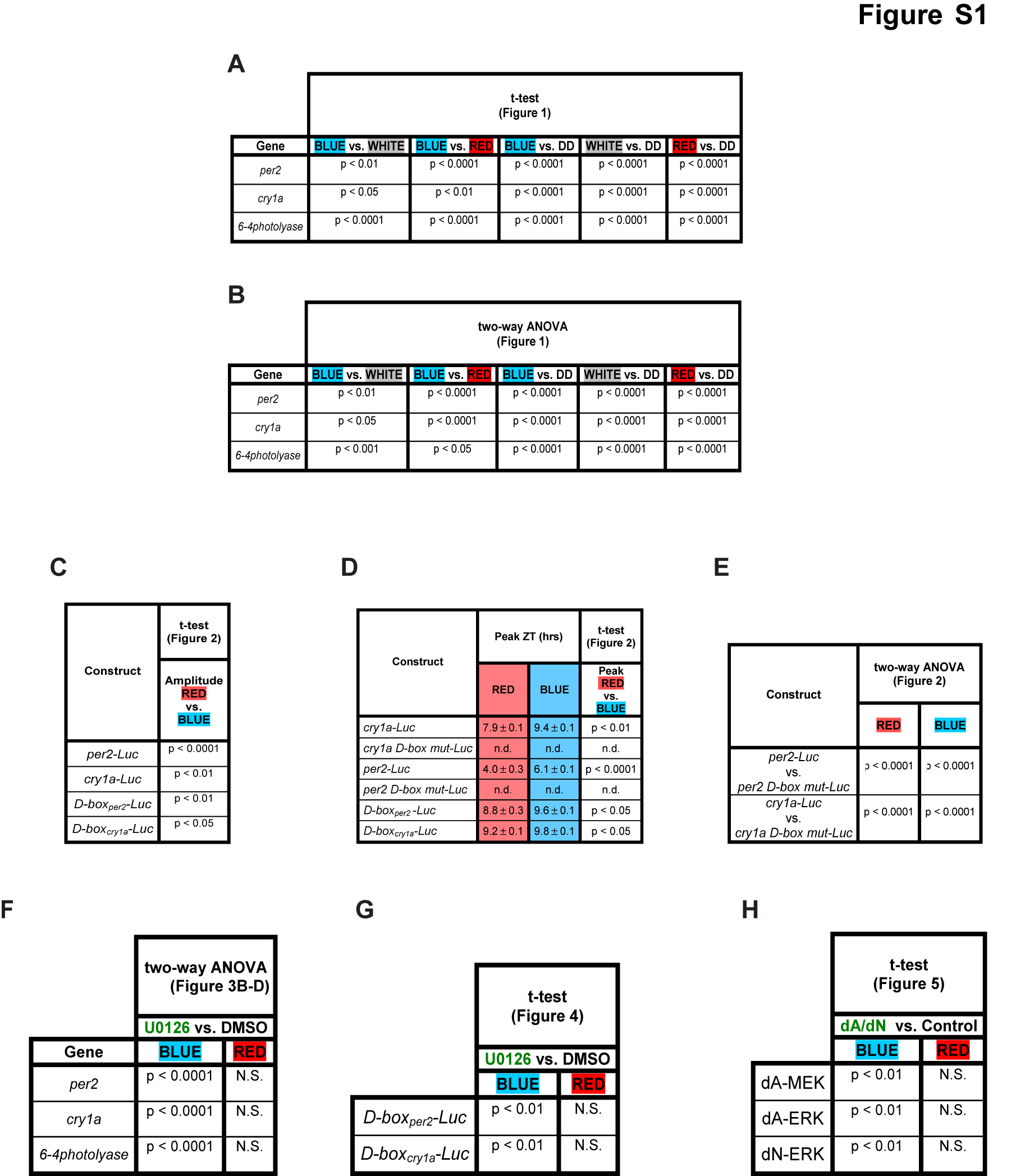

Supplement: Figure S1 — (A–H) Summary of statistical analysis. The panels represent the results of statistical analysis for all experiments. The relevant figure and the type of statistical test performed are indicated above each panel. A colour code illustrates the type of illumination used and the names of genes, constructs or treatments are indicated in each panel. Statistically significant was considered as p<0.05. No Significant difference is indicated by N. S. In panel D, peak time values were calculated using Ritme software (Antoni Diez-Noguera, University of Barcelona) and n.d. indicates “not-determined” peak values. (TIF) [file pone.0067858.s001.tif]

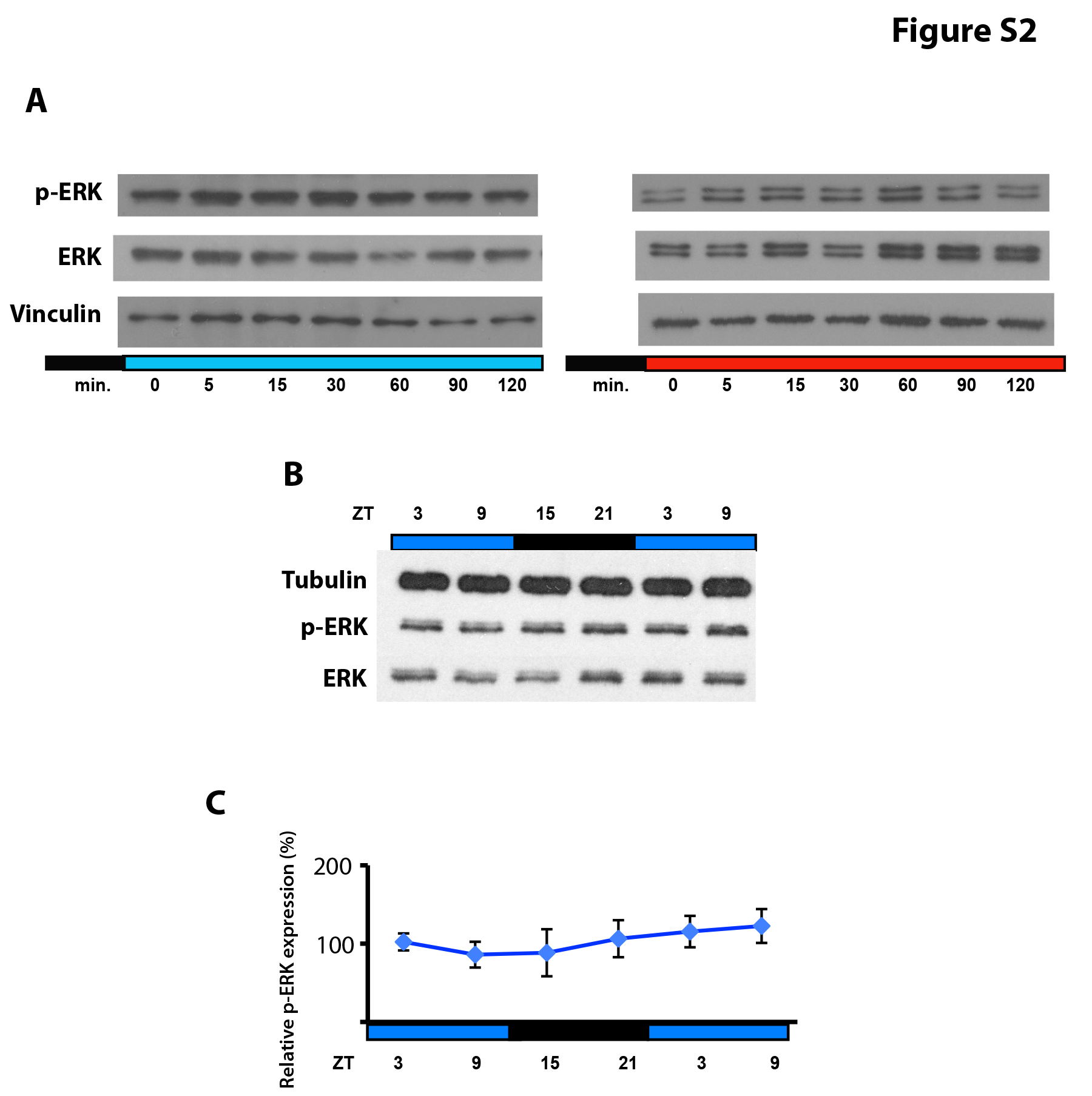

Supplement: Figure S2 — Western blot analysis of phospho-ERK under blue and red light. Representative western blots of endogenous ERK (ERK), phospho-ERK (p-ERK) and Vinculin or α-Tubulin in PAC-2 cells during (A) 2 hours of either blue or red light exposure and (B) exposure for 36 hours to a 12 hours blue; 12 hours dark LD cycle. In panel A, the duration of light exposure of each sample is indicated in minutes (mins). These blots are representative of six independent experiments and the final quantification is presented graphically in Figure 3A. In panel B, the time points are indicated as zeitgeber times (ZT, where ZT0 represents lights on and ZT12 represents lights off). Note that the p-ERK and ERK antibodies both recognize the two forms of ERK (p42 and p44). Thus, with these antibodies two bands are more or less well resolved depending on the duration of electrophoresis. Levels of Vinculin or α-Tubulin were used as loading controls for each western blot. Blue and red bars above or below each blot represent the wavelengths and duration of light exposure while black bars indicate darkness. (C) Quantification of relative phospho-ERK levels from three independent experiments performed as in panel B. No significant circadian oscillation was observed as tested by Cosinor analysis (COSINOR v3.0.2 software, Antoni Diez-Noguera, University of Barcelona). (TIF) [file pone.0067858.s002.tif]

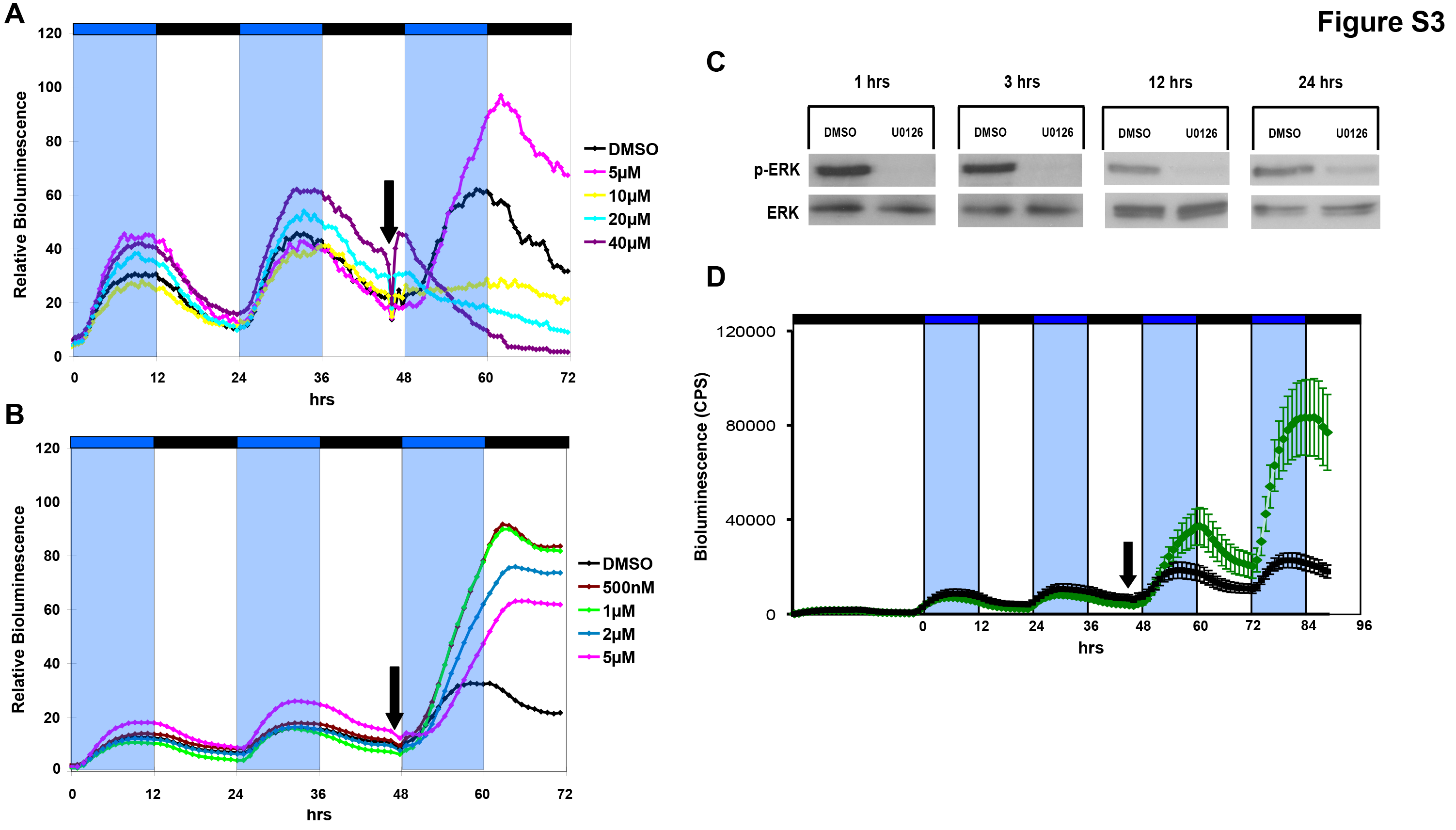

Supplement: Figure S3 — Inhibition of ERK phosphorylation by U0126 treatment. (A–B) Real time bioluminescence assays of PAC-2 cells transfected with D-boxcry1a-Luc and treated with a range of different U0126 concentrations or a DMSO control. The color code for each treatment is depicted in the key for the panels. The black arrows indicate the start of treatments. Relative bioluminescence is plotted on the y-axis and time (hrs) on the x-axis. Each time-point represents the mean of three independent experiments. Blue and black bars above the panels represent the different lighting conditions. For clarity, blue and white background also indicates the blue light and dark periods, respectively. (C) Representative western blots of endogenous phospho-ERK (p-ERK) and ERK levels in PAC-2 cells following 1 hr, 3 hrs, 12 hrs or 24 hrs of incubation with 1 µM U0126 or DMSO (control) and under DD conditions. (D) Real time bioluminescence assays of PAC-2 cells transfected with D-boxcry1a-Luc, in the presence (green trace) or absence (black trace) of the selected dose of U0126 (1 µM). The black arrow indicates the start of 48 hours of U0126 treatment. Relative bioluminescence is plotted on the y-axis and time from the first exposure to blue light (hrs) on the x-axis. Each time-point represents the mean of three independent experiments +/−SD. Periods of exposure to darkness or blue light are indicated as described for panels A and B. (TIF) [file pone.0067858.s003.tif]

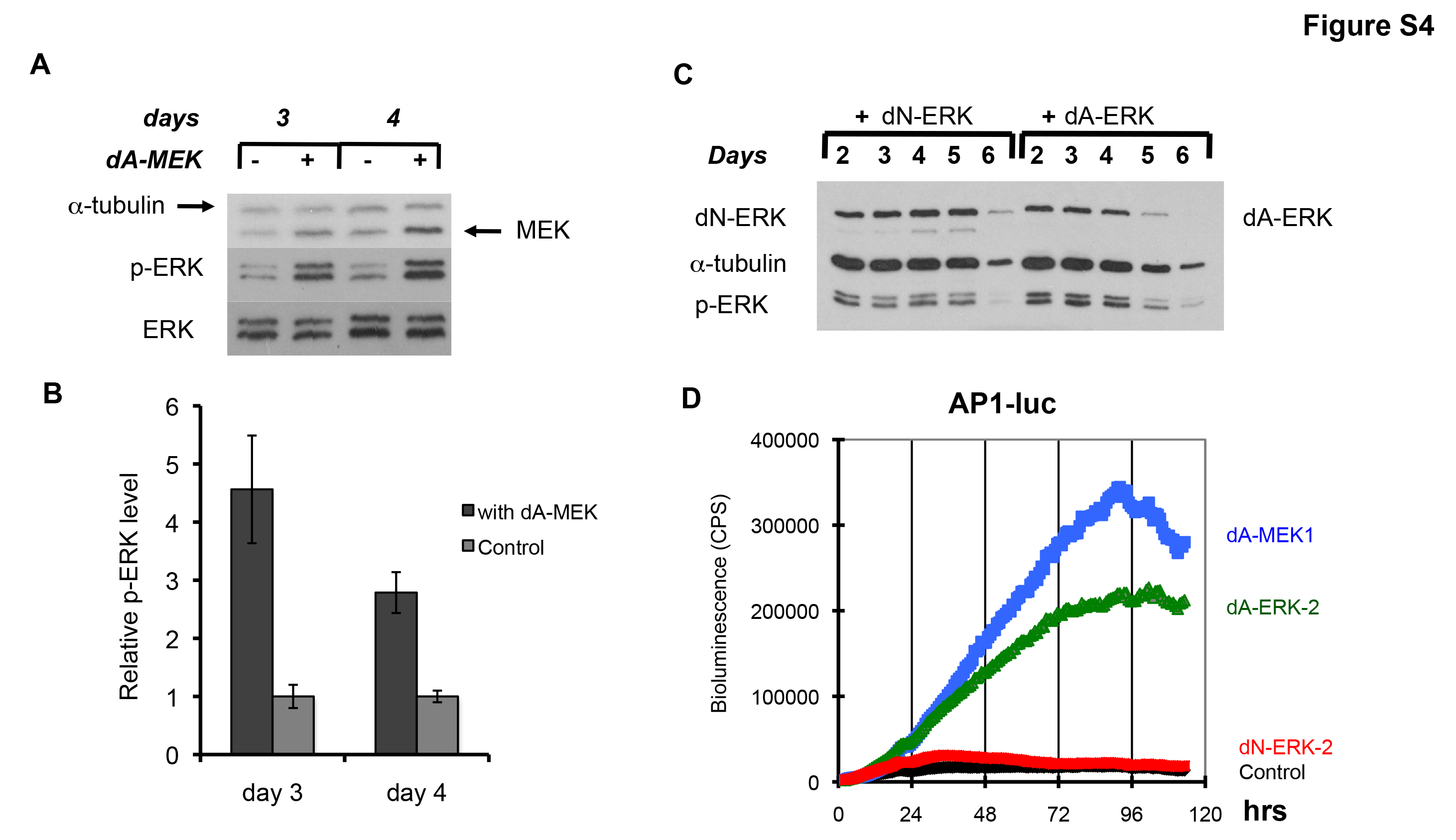

Supplement: Figure S4 — Characterization of dominant active and dominant negative ERK and MEK. (A) Representative western blot of endogenous ERK phosphorylation (p-ERK) levels in PAC-2 cells sampled at the same time point each day during the third and fourth day after transfection with dA-MEK (+) or with an empty expression vector (−) (control). Western blot analysis of MEK, ERK and α-tubulin in the same extracts are also shown as controls. Note that in the presence of dA-MEK, the level of immunoreactive MEK protein is increased since the electrophoretic mobility of the recombinant and endogenous MEK proteins are identical (see materials and methods for details of the recombinant MEK protein). (B) Quantification of the western analysis in panel A, performed in triplicate with phospho-ERK values normalized using endogenous ERK levels. The dark and light grey bars represent phospho-ERK levels in the presence and absence (control) of dA-MEK respectively. Relative ERK phosphorylation levels are plotted on the y-axis where levels in the control samples were set arbitrarily as 1. Values are plotted as the means of three independent experiments +/−SD. The levels of phospho-ERK are significantly increased in the presence of the dA-MEK form for both days (p<0.001, t-test). (C) The expression of the ERK-MEK fusion-proteins produced by dA-ERK and dN-ERK in cell extracts prepared at the same time each day, from the second to the sixth day following transfection was detected by western blot analysis using phospho-ERK (p-ERK) antibodies. (Note the high molecular weight of these fusion proteins with respect to the endogenous phospho-ERK protein visible in the same membrane, see materials and methods for precise details). As a loading control, the membrane was also incubated with an anti α-tubulin antibody. Note that by the sixth day, less concentrated protein extracts were recovered due to decreasing viability of the transfected cells. (D) Real time bioluminescence assays of PAC-2 cells transfected with [file pone.0067858.s004.tif]
