# Supplementary material for: ERK Signaling Regulates Light-Induced Gene Expression via D-Box Enhancers in a Differential, Wavelength-Dependent Manner
Source: PLoS One. 2013 Jun 26;8(6):e67858. doi: 10.1371/journal.pone.0067858 (PMC3694018; doi:10.1371/journal.pone.0067858)
Supplement: Table S1 — qRT-PCR primer sequences. (DOC) [file pone.0067858.s005.doc]

**Table S1**

**qRT-PCR Primers**

| **Gene** | **Primer Sequence** |
| --- | --- |
| *per2* | Forward: 5’- GCTTCACCACACCATACAGG -3’  Reverse: 5’- GTCTGACGGGGACGAGTCT -3’ |
| *cry1a* | Forward : 5’- TCCGCTGTGTGTACATCCTC -3’  Reverse : 5’- CAAACACTGCAGCAAAAACC -3’ |
| *6-4photolyase* | Forward: 5’- AATGGCAAGACTCCCATGAC -3’  Reverse: 5’- GTGGCCCTAAGGATGACGTA -3’ |
| * *β*-actin* | Forward : 5’- GCCTGACGGACAGGTCAT -3’  Reverse : 5’- ACCGCAAGATTCCATACCC -3’ |
